# Incidental, Solitary, and Unilateral Adrenal Metastasis as the Initial Manifestation of Lung Adenocarcinoma

**DOI:** 10.7759/cureus.32628

**Published:** 2022-12-17

**Authors:** Hadeel K Al Fares, Sierra Abdullaj, Neriman Gokden, Lakshmi P Menon

**Affiliations:** 1 Internal Medicine, University of Arkansas for Medical Sciences, Little Rock, USA; 2 Pathology, University of Arkansas for Medical Sciences, Little Rock, USA; 3 Endocrinology and Metabolism, Internal Medicine, University of Arkansas for Medical Sciences, Little Rock, USA

**Keywords:** metastatic adenocarcinoma, lung adenocarcinoma, adrenal biopsy, adrenal metastasis, adrenal incidentaloma

## Abstract

An adrenal incidentaloma is an adrenal mass ≥ 1 cm in size discovered on imaging performed for indications other than suspected adrenal disease. It has variable etiologies, which can be benign or malignant, including primary or metastatic disease. We present a rare case of metastatic lung adenocarcinoma with isolated unilateral adrenal metastases, presenting as an adrenal incidentaloma in an asymptomatic patient with no known history of malignancy. A 76-year-old man with a past medical history of chronic obstructive pulmonary disease (COPD) and heavy tobacco use was admitted for the evaluation and treatment of pneumonia. He was found to have an incidental 4.6 cm unilateral adrenal mass on his CT chest. He underwent a workup for the mass, including further imaging studies that were indeterminate and a hormonal workup that concluded that the mass was nonfunctional. Due to the patient’s comorbidities, it was determined that he was not a surgical candidate. A multidisciplinary team recommended a biopsy, which revealed metastatic lung adenocarcinoma. The primary lung cancer was located using positron emission tomography with 2-deoxy-2-(fluorine-18) fluoro-D-glucose combined with computed tomography (F-FDG-PET/CT). The patient was evaluated by an oncology service and started on chemotherapy. In this case report, we discuss the approach for evaluating adrenal incidentalomas as well as the role the biopsy has in this process based on a literature review. In addition, we draw a comparison between our case and similar cases in the literature while highlighting the differences that make this case unique.

## Introduction

An adrenal incidentaloma is an asymptomatic adrenal mass >1 cm in size, detected incidentally on imaging that was performed for another indication, without the suspected adrenal disease [1]. It affects almost 2% of the general population and 7% of those over 70 years of age [2]. Most adrenal incidentalomas are benign; however, they can have a malignant etiology, such as adrenocortical carcinoma or metastases [3]. The adrenal glands are a common site for metastases to spread due to their rich blood supply, with lung cancer being the most common primary site (35%), followed by the stomach (14%), the esophagus (12%), and the liver or bile ducts (10%) [4,5]. Most adrenal metastases are bilateral, and by the time they are discovered, the primary malignancy is usually already known. An adrenal incidentaloma, being the initial presentation of extra-adrenal malignancy, is extremely rare.

Here, we report the case of an asymptomatic patient with no known history of cancer who presented with a unilateral adrenal incidentaloma. Imaging was indeterminate; a biopsy revealed metastatic lung adenocarcinoma, after which the primary malignancy was located using a positron emission tomography with 2-deoxy-2-(fluorine-18) fluoro-D-glucose combined with a computed tomography (F-FDG-PET/CT) scan. The patient was subsequently diagnosed with oligometastatic lung adenocarcinoma, as metastases were limited to a single adrenal gland.

## Case presentation

A 76-year-old man with a history of coronary artery disease, congestive heart failure, obstructive sleep apnea, hypertension, peripheral vascular disease, hyperlipidemia, chronic obstructive pulmonary disease (COPD), and a history of heavy tobacco use presented to an outside hospital with a four-day history of cough, shortness of breath, and wheezing. CT chest without contrast revealed bilateral lower lobe pneumonia, diffuse emphysematous changes, and an incidental finding of a 3.5 x 2.9 x 4.6 cm left adrenal mass. The adrenal mass was not present on previous CT imaging from three years ago. He was treated with broad-spectrum antibiotics for pneumonia with clinical improvement. He had a 50-pack tobacco use history per year and quit smoking following this hospitalization.

On a repeat CT chest with contrast two months later, the adrenal mass measured 4.1 x 3.4 x 6.7 cm, with heterogeneity and high pre-contrast density. The imaging characteristics, along with the interval increase in size, were suspicious of malignancy. Of note, this imaging showed preexisting pulmonary emphysema with a 6 mm nodule in the superior segment of the left lower lobe that was stable when compared to imaging from three years ago; the imaging also showed improved consolidation in the bilateral lower lobes. He was started on steroids for secondary organizing pneumonia.

**Figure 1:**
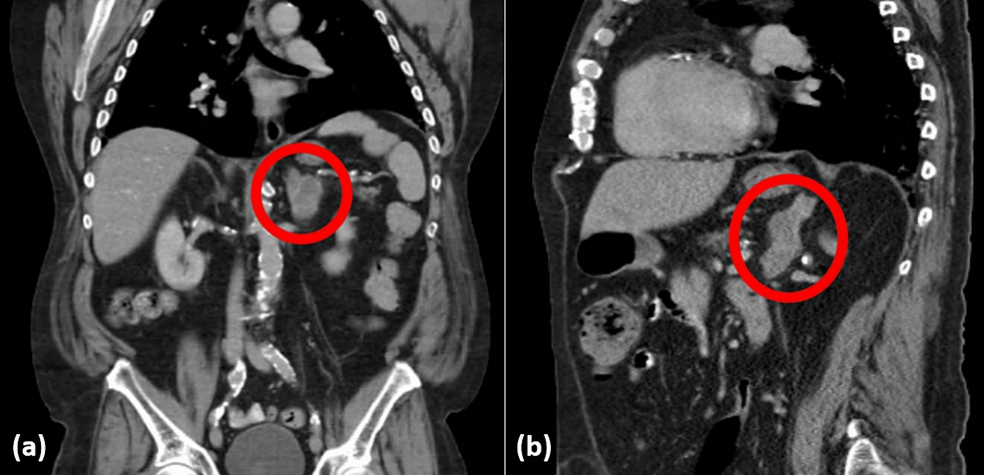
A multilobulated, heterogeneously enhancing left adrenal nodule can be seen in (a) the coronal section and (b) the sagittal section, measuring 2.4 x 3.8 x 6.3 cm (AP x TV x CC). AP: anteroposterior; TR: transverse; CC: craniocaudal

The patient was referred to the urology clinic for an enlarging left-sided adrenal mass. A repeat CT of the chest, abdomen, and pelvis on the day of the initial visit with urology demonstrated that the left adrenal nodule was stable in size (6.3 cm) compared to the second scan done three months ago (Figure 1). The pre-contrast density ranged from 17 to 27 Hounsfield units (HU), with an absolute washout of 84.6% and a relative washout of 28.2%, both of which were indeterminate. Scattered emphysematous changes were present throughout both lungs, with a 6 mm soft tissue nodule within the right middle lobe and a 6 mm soft tissue nodule within the superior segment of the left lower lobe. A few prominent mediastinal lymph nodes were seen, measuring up to 12 mm in the right paratracheal region.

At that time, his shortness of breath had improved since his hospitalization, but his physical activity remained significantly limited due to his chronic cardiopulmonary symptoms. He did not have any weight loss, fevers, chills, or gross hematuria. He was found to be a poor surgical candidate and was referred to endocrinology for a hormonal work-up, with a repeat CT scan planned in six weeks. The work-up revealed a normal complete blood count (CBC) and basic metabolic panel (BMP) (potassium 4.2mmol/L, creatinine 1.2mg/dl), cortisol of 7 ug/dl (5-23 ug/dl), adrenocorticotropic hormone (ACTH): 12.2 pg/ml (7.5-63.3 pg/ml), dehydroepiandrosterone sulfate (DHEAS): 13 ug/dl (28-175 ug/dl), plasma metanephrines: 0.16 nmol/L (0.0-0.49 nmol/L), and plasma renin: 7.4 ng/ml/hr (0.5-4.0 ng/ml/hr). These normal values ruled out Cushing’s syndrome, primary hyperaldosteronism, and pheochromocytoma, indicating that the nodule is nonfunctional.

**Figure 2:**
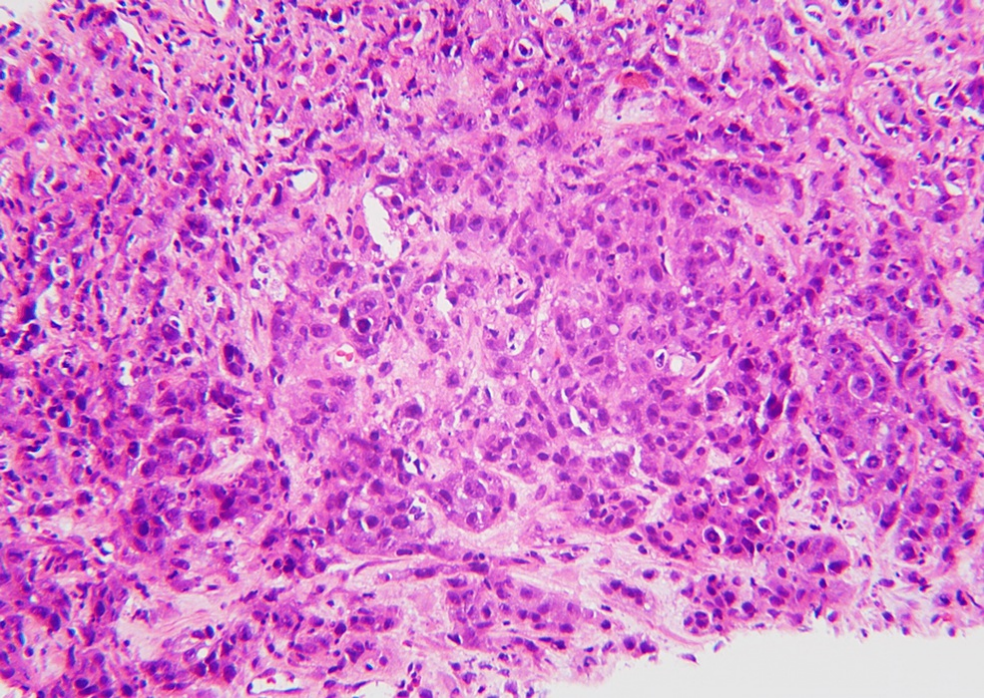
With hematoxylin and eosin stain at 20x magnification, tumor cells with large, hyperchromatic nuclei, prominent nucleoli, moderate eosinophilic cytoplasm, and brisk mitotic activity are visible.

**Figure 3:**
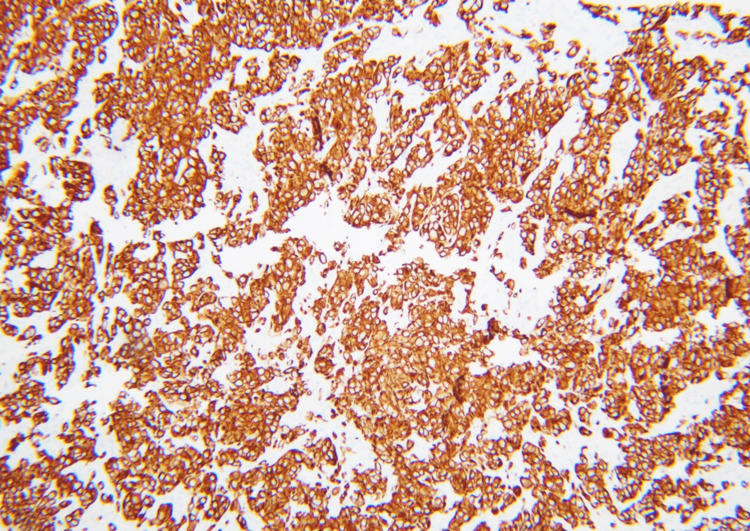
With the cytokeratin 7 stain, tumor cells demonstrate diffuse positive cytoplasmic staining.

**Figure 4:**
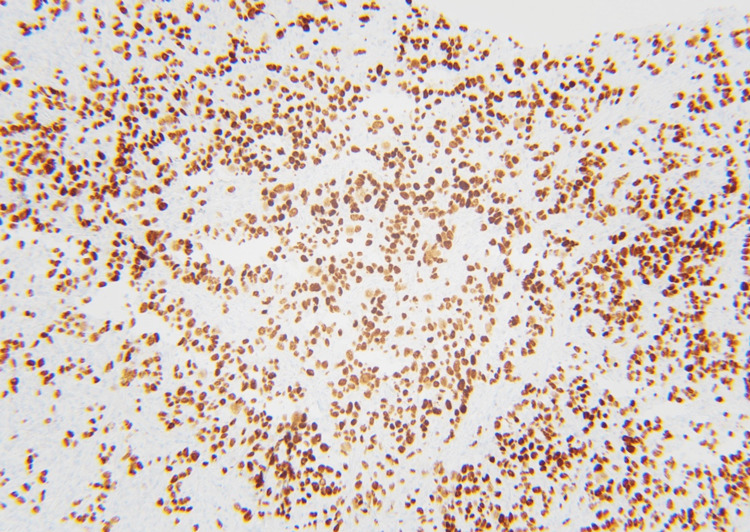
Thyroid transcription factor-1 staining reveals diffuse positive cytoplasmic staining in tumor cells, indicating a lung primary non-small cell carcinoma.

A follow-up CT scan with the renal mass protocol, two months after the urology appointment, revealed that the mass had remained stable in size. At the tumor board meeting, the decision was made to get further assessment via an F-FDG PET/CT scan followed by an adrenal mass biopsy due to the indeterminate CT findings and the patient’s poor surgical candidacy. The patient was initially unable to get a PET-CT scan due to insurance issues, so he underwent a left adrenal biopsy. Pathology revealed a metastatic carcinoma. The immunohistochemical panel was performed, and it showed tumor cells to be diffusely and strongly positive for cytokeratin 7 (CK7) and thyroid transcription factor 1 (TTF-1), while negative for cytokeratin 20 (CK20), melan-A, inhibin, and S100. Based on the morphology and immunohistochemical profile of the tumor cells, a metastatic adenocarcinoma most likely from the lung primary was favored (Figures 2-4).

**Figure 5:**
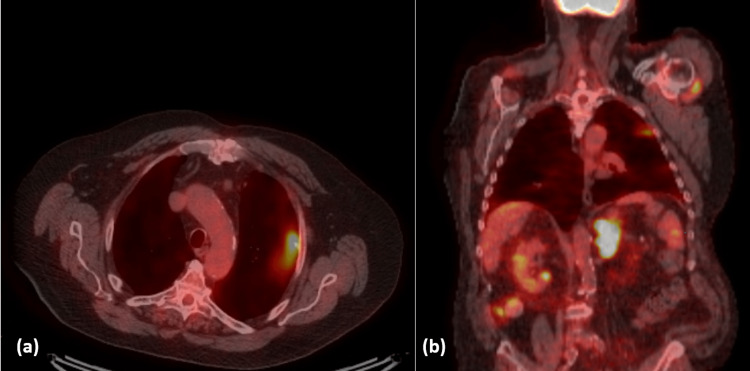
(a): Moderate to intense FDG activity can be seen fusing to left lung upper lobe peripheral reticulonodular changes, measured by SUV max 4.6. (b): Additional intense FDG activity fusing to the left renal mass, measuring SUV max 16.2, can be visualized along with the previously mentioned FDG activity seen in the left lung upper lobe.

The patient was eventually able to undergo an F-FDG PET/CT scan, which revealed intense FDG activity fusing to the left renal mass, measuring standardized uptake value (SUV) max 16.2, consistent with the biopsy-proven disease, and moderate to intense FDG activity fusing to left lung upper lobe peripheral reticulonodular changes, measuring SUV max 4.6 (Figure 5). The patient was diagnosed with stage IVA lung adenocarcinoma. Further staging with a brain MRI was obtained and was negative; the patient had oligometastatic non-small cell lung cancer (NSCLC), limited to the left adrenal gland.

Molecular testing of the biopsy specimen by the 22C3 assay revealed a programmed cell death ligand 1 (PD-L1) score of 50%. Based on these results, Keytruda (pembrolizumab) was recommended during the following oncology visit. After that, the patient chose to transfer his care to an oncologist closer to his home to receive this treatment.

## Discussion

We reported a rare case of metastatic lung adenocarcinoma with isolated unilateral adrenal metastases, presenting initially as an asymptomatic adrenal incidentaloma. The patient did not have a prior history of lung cancer but had a long history of smoking and COPD. He had two preexisting stable 6 mm nodules on the CT chest that were not suspicious of malignancy, along with emphysematous changes and a few prominent mediastinal paratracheal lymph nodes. In this case, the adrenal incidentaloma was found on a CT chest, and an endocrine workup showed it was nonfunctional. The patient was not a surgical candidate. The diagnosis was established via a biopsy of the adrenal mass, which revealed lung adenocarcinoma. An FDG PET scan detected intense FDG activity in the adrenal incidentaloma and in the upper lobe of the left lung, where the location of primary cancer is likely, which is a different area from where the preexisting lung nodules were located.

Most adrenal incidentalomas are nonfunctioning adrenocortical adenomas; other etiologies include but are not limited to adrenocortical carcinoma, pheochromocytoma, aldosterone- or cortisol-producing adenomas, or metastases. It is recommended to establish whether the mass is benign or malignant at the time of the initial detection of the incidentaloma [3]. The initial imaging of choice is a non-contrast CT scan of the abdomen; if it shows a homogeneous adrenal mass with an attenuation of ≤10 Hounsfield units, this indicates that the mass is benign in etiology, and hence no further imaging is required [1,2]. Nevertheless, 30% of adrenocortical adenomas have an attenuation of >10 HU due to poor lipid content, a feature also seen in malignant lesions and pheochromocytomas; in such cases, a non-enhanced CT alone is unreliable [2,6]. Therefore, for adrenal lesions with an attenuation of >10 HU, additional imaging with a contrast-enhanced CT with an adrenal washout protocol can be performed. An absolute washout of >60% or a relative washout of >40% indicates an adrenocortical adenoma [7]. This modality was shown to have a high sensitivity (96%) and specificity (95%) for differentiating between adrenal adenomas and non-adenomas based on a prospective study that included 50 patients with adrenal masses [8]. If the imaging features remain indeterminate and the hormonal workup suggests the mass is nonfunctional, the patient’s clinical condition and presentation are taken into consideration by a multidisciplinary team. The team then might recommend additional imaging with another modality, repeat imaging in six to 12 months, or surgery. In patients with a history of extra-adrenal malignancy, FDG-PET/CT can be performed if the mass remains indeterminate on the previously mentioned imaging modalities [1]. As for adrenal biopsies, a systematic review concluded that they are most useful in diagnosing adrenal metastases; therefore, an adrenal biopsy is generally not recommended unless there is a history of current or prior extra-adrenal malignancy and if the biopsy result is expected to alter management [1,9]. Pheochromocytoma should also be excluded before performing a biopsy to prevent the development of hypertensive crises. In an adrenal biopsy, a metastatic adenocarcinoma diagnosis would require a panel of immunohistochemical stains. In most cases, a panel of CK7, CK20, TTT-1, inhibin, S100, and melan-A would be helpful to point to a primary site. In this case, the tumor cells were positive for cytokeratin 7 (CK7) and thyroid transcription factor-1 (TTF-1). For CK7, a previous study has shown that the expression of CK7 is seen in the majority of cases of carcinoma, except for those originating from the colon, prostate, kidney, and thymus. A positive CK7 can also exclude adrenocortical carcinoma, as the tumor cells do not express CK7 and inhibin [10]. A metastatic melanoma was ruled out by the negative staining for S100 and melan-A. As for TTF-1, it is detected in up to 95% of antibody-drug conjugates (ADC) of the lung and is commonly used to distinguish primary lung adenocarcinoma from tumors from other sources [11].

As for the hormonal workup of adrenal incidentalomas, it is recommended that all patients undergo a low-dose dexamethasone suppression test to exclude autonomous cortisol secretion. It is also recommended to exclude pheochromocytoma in all patients by measurement of plasma metanephrines or urinary fractionated metanephrines. For patients with hypertension or hypokalemia, plasma aldosterone and plasma renin can be measured to exclude primary aldosteronism. In patients with clinical or imaging features of adrenocortical carcinoma, measurement of sex hormones is recommended [1].

In the case we presented above, the adrenal incidentaloma was found in an asymptomatic patient without a history of malignancy; a biopsy revealed metastatic lung adenocarcinoma; and an FDG PET scan identified the primary malignancy location in the lung, along with the isolated unilateral adrenal metastasis. In a literature review, a similar case was reported where a 67-year-old patient with no history of malignancy was being evaluated for a 4.6 cm adrenal incidentaloma that was found during a workup for nephrolithiasis. The mass was nonfunctional, and he eventually underwent an adrenalectomy, which revealed metastatic papillary thyroid cancer. A thyroid ultrasound later identified a nodule where the primary malignancy was located [12].

In a study that was done to examine the role of FNA in the workup of adrenal incidentalomas, data from 1715 patients referred for evaluation of suspected unknown primary cancer were retrospectively reviewed; 1639 patients were found to have malignancies, and the adrenal gland was involved in 95 patients (5.8%). Only four of the 95 patients (0.2% of the 1639 patients) had isolated adrenal metastases; three of them were found to have lung adenocarcinoma, and only one had a unilateral lesion, but in none of the cases was primary cancer located. It is noteworthy that none of these adrenal masses were true incidentalomas, as all four had abdominal pain, and three of them had anorexia. The study concluded that metastatic cancer presenting as a true incidentaloma was extremely rare (0 out of 1639), so FNA was not recommended as part of the routine workup for a patient presenting with a true unilateral adrenal incidentaloma without a prior history of malignancy [13].

The case we are reporting is unique in that the adrenal mass was a true incidentaloma as the patient was asymptomatic; it was also unilateral, while the majority of adrenal metastases are bilateral [13]. An F-FDG PET/CT revealed that the metastatic disease was limited to the adrenal gland without other organ involvement, which is similar in comparison to the four cases mentioned in the study above [13]. However, unlike the other four cases, the PET scan performed on the patient, in this case, was able to detect primary cancer, whereas the primary malignancy was never found.

It is also notable that the two preexisting 6 mm nodules found on the CT chest were located in the superior segment of the left lower lobe and in the right middle lobe, while the primary malignancy was located in the upper lobe of the left lung, where the F-FDG PET/CT scan detected intense FDG activity.

## Conclusions

Due to the rarity of adrenal metastases presenting as unilateral adrenal incidentalomas, previous studies showed no benefit of adrenal biopsies in such cases where the patient lacks any history of prior or current malignancy, and has no symptoms suggestive of occult malignancy. However, in our case, obtaining a biopsy of the adrenal incidentaloma eventually led to a diagnosis of metastatic primary lung adenocarcinoma.
